# Struktur- und Prozessevaluation komplexer Interventionen in der Schmerztherapie: Beschreibung eines methodischen Ansatzes am Beispiel von POET-Pain

**DOI:** 10.1007/s00482-024-00850-w

**Published:** 2024-12-10

**Authors:** Irmela Gnass, Stefanie Berger, Nina Schürholz, Ulrike Kaiser, Axel Schäfer, Alexander Schnabel, Esther Pogatzki-Zahn, Nadja Nestler

**Affiliations:** 1 Institut für Pflegewissenschaft und -praxis, Paracelsus Medizinische Privatuniversität, Strubergasse 21, 5020 Salzburg, Österreich; 2 Klinik für Anästhesiologie und Intensivmedizin, Universitätsklinikum Schleswig-Holstein, Campus Lübeck, Schleswig-Holstein, Deutschland; 3 Fakultät Soziale Arbeit und Gesundheit, Hochschule für angewandte Wissenschaft und Kunst Hildesheim/Holzminden/Göttingen, Hildesheim, Deutschland; 4 Klinik für Anästhesiologie, operative Intensivmedizin und Schmerztherapie, Universitätsklinikum Münster, Münster, Deutschland

**Keywords:** Versorgungsforschung, Mixed-Methods-Design, Neue Versorgungsformen, Transitional Pain Service, Chronischer postoperativer Schmerz, Health services research, Mixed methods design, New healthcare interventions, Transitional pain service, Postoperative pain, chronic

## Abstract

Zur Bewertung der Versorgungsqualität, insbesondere bei neuen Versorgungsformen (nVF), werden die zu erbringenden Gesundheitsleistungen vorab definiert und dokumentiert. Das vorgestellte Explanatory-Sequential-Mixed-Methods-Design kombiniert quantitative und qualitative Datenerhebungen, und seine Analyse ermöglicht ein vertieftes Verständnis einer nVF. Am Beispiel des Projekts POET-Pain, das die Wirkung eines perioperativen „Transitional Pain Service“ (TPS) untersucht, wird die methodische Anwendung des Explanatory-Sequential-Mixed-Methods-Designs demonstriert, um die Struktur- und Prozessevaluation der nVF (hier TPS) vorzustellen und deren Einfluss auf die Versorgungsqualität zu verstehen. Das vorgestellte Mixed-methods-Design ermöglicht es, die Ergebnisse der quantitativen Phase anhand qualitativer Daten vertiefend zu interpretieren und zu erweitern, was zu einem umfänglichen Verständnis des Gegenstands (zweite Säule der Versorgungsforschung) führt.

**Zusatzmaterial online:**

Die Online-Version dieses Beitrags (10.1007/s00482-024-00850-w) enthält den Erhebungsbogen und die Merkmalsverteilung.

## Abstract (en)

To evaluate the quality of care, particularly in the case of new forms of healthcare interventions, the healthcare services to be provided are defined and documented in advance. The presented explanatory sequential mixed methods design combines quantitative and qualitative data collection and the analysis enables a deeper understanding of a new healthcare intervention. Using the example of the POET-Pain project, which investigates the effect of a perioperative transitional pain service (TPS), the methodological application of the explanatory sequential mixed methods design is demonstrated in order to present the structural and process evaluation of the new healthcare intervention (in this case TPS) and to understand its influence on the quality of care. The mixed methods design presented enables the results of the quantitative phase to be interpreted and expanded in depth using qualitative data, which leads to a comprehensive understanding of the subject matter (second pillar of health services research).

Ein zentrales Konzept in der Versorgungsforschung ist die Versorgungsqualität, die durch verschiedene Faktoren beeinflusst wird. Zur Bewertung der Versorgungsqualität beispielsweise neuer Versorgungsformen (nVF) werden die Leistungen, die Patientinnen und Patienten erhalten, vorab definiert und dokumentiert. Dieses Vorgehen ermöglicht die Beschreibung, Erklärung, Gestaltung und nach der Evaluation ein Verständnis dafür, wie professionsspezifische und interprofessionell abgestimmte Leistungen zur Versorgungsqualität beitragen [28, 33]. Ein bewährtes Modell zur Evaluation der Versorgungsqualität ist das Donabedian-Konzept, das die Versorgungsqualität in drei Hauptkomponenten unterteilt [8]:

- Struktur
- Prozess
- Ergebnis

Strukturen umfassen dabei die physischen und organisatorischen Rahmenbedingungen, die die Bereitstellung von Gesundheitsdienstleistungen ermöglichen (Kontext der Gesundheitsleistung). Prozesse beziehen sich auf die spezifischen Maßnahmen und Aktivitäten, die während der Versorgung durchgeführt werden (Intervention). Ergebnisse schließlich sind die Endpunkte der Versorgung, sprich wie sich die Gesundheitsergebnisse bei den Patientinnen und Patienten entwickeln. Daher kann in Anlehnung an das Donabedian-Konzept mit der Analyse von Strukturen und Prozessen ein umfassender Blick auf Faktoren genommen werden, die Wirkung auf die Implementierung und Versorgungsqualität nehmen könnten. Diese Art der Untersuchung im Kontext von Versorgungsforschung kann Einblicke in die Stärken und Schwächen der Gesundheitsleistung bieten und somit zur kontinuierlichen Verbesserung der Versorgungsqualität beitragen [9].

## Doppelte Komplexität – Kontext und Intervention

Wie oben ausgeführt, weisen der Kontext der Gesundheitsleistung und die Intervention jeweils eine eigene Komplexität auf, die bei Umsetzung der nVF eine doppelte Komplexität entwickeln, die im Folgenden detaillierter betrachtet wird.

Zum Kontext der Gesundheitsleistung gehört einrichtungsspezifisch die Zusammenarbeit bzw. Zusammensetzung der Fachkräfte in einer Abteilung und die Umsetzung einer evidenzbasierten und auch patientinnen- bzw. patientenzentrierten Versorgungspraxis. Somit sind die klinik- oder abteilungseigene Organisationskultur, die Personalressourcen und die Ausstattung im jeweiligen Bereich als explizite Komponenten für die Umsetzung einer Intervention von Bedeutung. Implizite Komplexität, beispielsweise Werte, Normen und Überzeugungen innerhalb einer Klinik, ist wiederum prägend für die Bereitschaft und Fähigkeit des Personals, neue Interventionen zu akzeptieren und umzusetzen. Ferner sind die Verfügbarkeit und Qualifikation des Personals entscheidend für die Umsetzung von Interventionen und Innovationen.

Der Kontext der Gesundheitsleistung und die Intervention weisen jeweils eine eigene Komplexität auf

Die Intervention kann aus einzelnen Maßnahmen (beispielsweise der Verabreichung eines Schmerzmedikaments) bestehen, aber auch komplexe, mehrstufige Behandlungspläne (wie die Versorgung durch einen Akutschmerzdienst) umfassen. Wichtig ist, dass die Entwicklung der Interventionen auf wissenschaftlichen Erkenntnissen und klinischer Erfahrung hinsichtlich der vielfältigen Gesundheitsbedarfe von Patientinnen und Patienten beruht und in weiterer Folge angepasst wird [36].

Da die Umsetzbarkeit einer komplexen Intervention im Sinne der doppelten Komplexität mehrere Perspektiven beinhaltet, können neben der Erfassung von Strukturen und Prozessen auch zu deren Auswirkungen bei Anwendenden (Versorgungsteams) und Empfangenden (Patientinnen und Patienten) wichtige Erkenntnisse zur Umsetzung und Nutzung der Intervention erfasst werden. Die Beschreibung dieser Perspektiven erfüllt somit auch das Konzept von Donabedian, das eine explizite Betrachtung von Strukturen und Prozessen für die Nachvollziehbarkeit der Ergebnisqualität bei neuen Versorgungsmodellen bzw. -formen fokussiert [26].

### Zielsetzung der Struktur- und Prozessevaluation

In der Annahme, dass eine nVF (komplexe Intervention) positive Effekte bei Patientinnen und Patienten mit einem spezifischen Versorgungsbedarf zeigt, besteht das Ziel einer Struktur- und Prozessevaluation darin, die Bedeutung unterschiedlicher möglicher Versorgungsaspekte zu identifizieren und zu beschreiben sowie versorgungsrelevante Erkenntnisse für die Überführung in die Regelversorgung zu erklären.

## Explanatory-Sequential-Mixed-Methods-Design

Für die Erfassung der unterschiedlichen Perspektiven auf die Strukturen und Prozesse im Kontext einer komplexen Intervention kann die Datenerhebung und -auswertung entlang eines Mixed-methods-Designs, hier explizit des Explanatory-Sequential-Mixed-Methods-Designs, Anwendung finden [6].

Das angewendete Explanatory-Sequential-Mixed-Methods-Design (Zwei-Phasen-Design) besteht aus Datenerhebung und -auswertung mit zwei aufeinanderfolgenden methodischen Schritten:

- Phase I: *quantitative* Datenerhebung/-auswertung
- Phase II: *qualitative* Datenerhebung/-auswertung

**Abb. 1:**
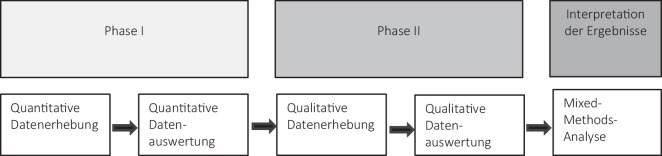
Explanatory-Sequential-Mixed-Methods-Design (Zwei-Phasen-Design)

Im letzten Schritt, der Mixed-methods-Analyse, erfolgt dann die Interpretation der Ergebnisse. Damit verdichten die Erkenntnisse aus der qualitativen Phase die Ergebnisse aus der quantitativen Phase. So soll eine umfängliche Beschreibung und Interpretation für die nVF vorliegen [6, 36]. Eine schematische Darstellung der gesamten Datenerhebung und -auswertung findet sich in Abb. 1. Die einzelnen Phasen und die Mixed-methods-Analyse werden im weiteren Verlauf an einem Beispiel detailliert erläutert.

## Beispiel POET-Pain

In der Versorgung von Patientinnen und Patienten, die sich einem operativen Eingriff unterziehen, wird neben vielen anderen diagnostischen und therapeutischen Maßnahmen immer auch ein Versorgungsschwerpunkt auf die Schmerztherapie gelegt [7]. Prävalenzzahlen zeigen, dass neben dem akuten Schmerz etwa 10 % der Patientinnen und Patienten in einem Zeitraum zwischen 6 und 12 Monaten nach Operation moderate bis schwere chronische postoperative Schmerzen („chronic postsurgical pain“ [CPSP]) haben [12, 31]. Der Fokus der perioperativen Schmerztherapie liegt daher zunehmend auf der Identifizierung jener Patientinnen und Patienten, bei denen sich möglicherweise nach einer Operation eine postoperative Schmerzchronifizierung entwickelt [16]. Aus vielen Observationsstudien sind Faktoren (beispielsweise Alter und Geschlecht) bekannt, die das Risiko der Entwicklung von CPSP nachweislich erhöhen können, wobei angenommen wird, dass die Patientinnen und Patienten von präventiven Therapieansätzen zur Vermeidung von CPSP profitieren könnten [25, 30, 32, 34, 35]. Die Faktoren sind vielschichtig, weshalb Präventionsansätze, die nach der Identifizierung von Risikopatientinnen und -patienten angewendet werden, die Mehrdimensionalität des Schmerzes auf Grundlage des biopsychosozialen Modells berücksichtigen [2, 11, 17].

Folgendes Vorgehen wird in Leuchtturmprojekten international praktiziert: Vor einer geplanten größeren Operation wird die Wahrscheinlichkeit für die Entwicklung von CPSP eingeschätzt [25, 30]. Patientinnen und Patienten mit einem erhöhten Risiko erhalten idealerweise eine perioperative präventive Behandlung durch ein interprofessionelles Team, den sogenannten „transitional pain service“ (TPS). Entsprechend der biopsychosozialen Komplexität, die zur Entstehung von CPSP führt, besteht dieses Team aus Ärztinnen/Ärzten, Pflegefachpersonen, Physiotherapeutinnen/Physiotherapeuten sowie Psychologinnen/Psychologen und gegebenenfalls weiteren Professionen [1, 17]. Das TPS-Team stimmt auf Basis professionsspezifischer Assessments gemeinsam die Behandlungsansätze vor dem Hintergrund des individuellen Versorgungsbedarfs ab und setzt die Behandlungspläne gemeinsam und mit den Patientinnen und Patienten über einen Zeitraum von der präoperativen Phase bis mehrere Wochen oder sogar Monate nach der Operation um [1, 3, 4].

Bisher fehlten randomisierte, kontrollierte multizentrische Studien, die nachweisen, dass ein solcher TPS einen Nutzen in Bezug auf die Entwicklung von CPSP hat. Deshalb wurde im Rahmen eines Innovationsfondsprojekts (Förderkennzeichen 01NVF19021; [13]) eine randomisierte, kontrollierte Studie (RCT) initiiert, die sich zum Ziel gesetzt hat, den Effekt eines TPS als nVF auf das Auftreten, die Entwicklung und die Ausprägung chronischer Schmerzen erstmalig zu untersuchen. Weitere Informationen zur registrierten RCT POET-Pain [10] sind auf der projekteigenen Internetseite zu finden (https://www.poet-pain.de); POET-Pain steht für „Prävention Operationsbedingter anhaltender Schmerzen durch die Einführung eines perioperativen Transitional Pain Service“. Im vorliegenden Beitrag dient die parallel zur RCT erfolgte methodische Anwendung des Explanatory-Sequential-Mixed-Methods-Designs im Projekt POET-Pain als Beispiel für eine Struktur- und Prozessevaluation im Sinne einer Begleiterhebung.

Die RCT, die im Rahmen des Projekts POET-Pain die Hauptstudie darstellt, untersucht die Effektivität einer besonderen Versorgung von Patientinnen und Patienten mit einem erhöhten Risiko für eine Schmerzchronifizierung nach einer Operation mittels eines TPS. In diese Studie wurden an 6 Kliniken knapp 2000 Patienten eingeschlossen. Kriterien für den Ein- und Ausschluss von Patientinnen und Patienten in die RCT POET-Pain finden sich unter der Registrierung des Projekts [10]; Einschlusskriterien sind unter anderem erwachsene Patientinnen und Patienten mit elektiver Operationsindikation und geplantem stationärem Aufenthalt von mindestens 2 Tagen. Im Rahmen der Studie wurde vor der Operation mithilfe von Fragebögen überprüft, ob bei einer Patientin/einem Patienten ein erhöhtes Risiko für die Entwicklung anhaltender Schmerzen nach einer Operation bestand. War dies der Fall, so wurde er/sie per Zufall („randomisiert“) entweder der Risikotherapiegruppe (mit Behandlung durch den TPS) oder der Risikokontrollgruppe (mit in der Klinik üblicher Behandlung) zugeordnet. Ergab sich aus dem Risikoscreening dagegen kein erhöhtes Risiko für anhaltende Schmerzen nach einer Operation, so wurden die entsprechenden Patientinnen und Patienten einer Nichtrisikokontrollgruppe (mit in der Klinik üblicher Behandlung) zugeteilt [10]. Die RCT aus POET-Pain ist allerdings nicht Thema dieses Beitrags und wird daher nicht weiter ausgeführt.

### Erhebungszeitpunkte Phase I und Phase II

**Abb. 2:**
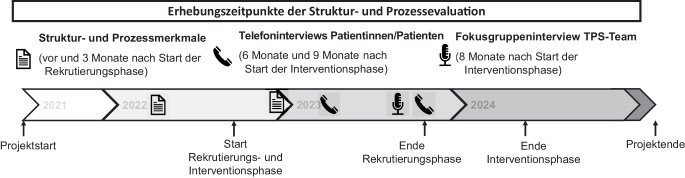
Erhebungszeitpunkte der Struktur- und Prozessevaluation am Beispiel POET-Pain. *TPS* „transitional pain service“

Die einzelnen Zeitpunkte der Datenerhebungen in Phase I und Phase II des Explanatory-Sequential-Mixed-Methods-Designs waren wie bereits erwähnt parallel zur RCT POET-Pain bzw. in diese eingebettet geplant (Abb. 2). Das Vorgehen bei den Datenerhebungen in den beiden Phasen und die jeweiligen Erhebungszeitpunkte werden im Folgenden detaillierter beschrieben.

#### Phase I – quantitative Datenerhebungen

In der Phase der quantitativen Datenerhebung stand die Beschreibung der Umsetzbarkeit der im Vorfeld definierten Strukturen und Prozesse des TPS der nVF in den einzelnen Kliniken im Fokus. Es sollte aufgezeigt werden, wie die Gesundheitsleistungen unter den Rahmenbedingungen der jeweiligen Klinik umgesetzt wurden.

Entlang der Fragestellungen *„Welche Strukturen werden bei der Umsetzung der nVF in den Kliniken eingesetzt?“* und *„Wie werden notwendige Prozesse der nVF organisiert?“* wurde ein strukturierter Erhebungsbogen entwickelt. Der inhaltliche Fokus lag auf den Merkmalen

- Professionen, Personalqualifikation/-umfänge,
- räumliche und technische Voraussetzungen,
- Organisation und Kommunikation im TPS,
- Regelungen zu TPS-Teamsitzungen und
- Regelungen zur Vermittlung der TPS-Therapieempfehlungen.

Der Erhebungsbogen (Fragebogen) wurde vorab im wissenschaftlichen Team von POET-Pain inhaltlich validiert. Die Erhebung der Daten erfolgte in allen 6 Kliniken zu 2 Messzeitpunkten (vor und 3 Monate nach Beginn der Rekrutierungsphase; Abb. 2), da in Anlehnung an die „normalisation process theory“ [27] davon auszugehen war, dass sich Veränderungen über die Zeit abzeichnen würden, die für die explizite Beschreibung zur Umsetzung einer Intervention bedeutsam sind.

**Abb. 3:**
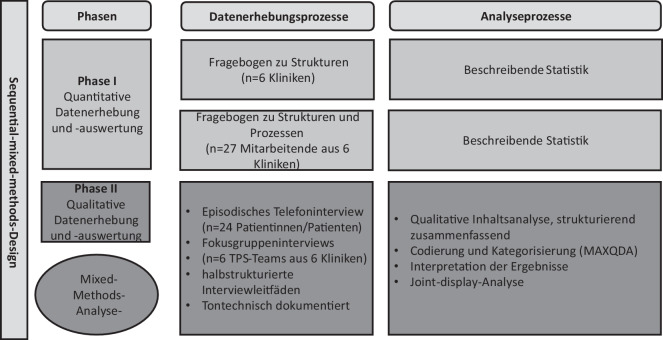
Prozessdiagramm – Explanatory-Sequential-Mixed-Methods-Design am Beispiel POET-Pain. *TPS* „transitional pain service“

Der Versand der Erhebungsbögen (Fragebögen) erfolgte per E‑Mail an die Studienkoordination in der jeweiligen Klinik, die dann die Verteilung an die jeweilig verantwortlichen Personen bzw. Professionen im TPS sicherstellte. Die Rücksendung an die Datenerhebenden war klinikintern so organisiert, dass für die Auswertung ausschließlich anonymisierte Daten vorlagen. Zum ersten Zeitpunkt wurden die Strukturen und zum zweiten Zeitpunkt die Strukturen und Prozesse der 6 Kliniken mit den Fragebögen erfasst (siehe Online-Zusatzmaterial 1: Erhebungsbögen). In beiden Erhebungsphasen wurden 27 Mitarbeitende aus den 6 eingeschlossenen Universitätskliniken befragt (Abb. 3).

Zum ersten Zeitpunkt wurden die Strukturen, zum zweiten Zeitpunkt Strukturen und Prozesse erfasst

Neben der Überprüfung der Datenqualität wurde zu beiden Erhebungszeitpunkten die Datenauswertung mit deskriptiver Statistik (absolute und relative Häufigkeiten) durchgeführt (Abb. 3).

#### Phase II – qualitative Datenerhebungen

Das Ziel von Phase II war es, mittels qualitativer Datenerhebungen ein vertieftes Verständnis für die nVF in Bezug auf die Umsetzung zu erhalten. Dazu wurde das Erleben aus der Perspektive *des TPS-Teams* und *der Patientinnen und Patienten* erhoben (Abb. 3).

##### Perspektive des TPS-Teams.

Entlang der Fragestellungen *„Wie erfolgt die Umsetzung der nVF?“, „Welche Arbeitsbedingungen/Ressourcen sind zur Umsetzung eines TPS erforderlich?“, „Wie arbeitet das TPS-Team interprofessionell zusammen?“* und *„Wie werden interprofessionelle Entscheidungen getroffen?“* wurde ein halbstandardisierter Interviewleitfaden entwickelt.

In Form eines Fokusgruppeninterviews [18] wurden die professionelle Expertise und das Erleben erhoben, welche relevant für die Beantwortung der Forschungsfragen waren; dabei wurden die Interviewten als Einzelpersonen wie auch als Gruppenmitglieder im TPS-Team angesprochen. In die Fokusgruppeninterviews waren in den 6 Kliniken ausschließlich Personen eingeschlossen worden, die im TPS-Team für die Umsetzung der nVF eingesetzt waren. Die Datenerhebung wurde 8 Monate nach Start der Interventionsphase durchgeführt (Abb. 2), womit die TPS-Teams bereits Patientinnen und Patienten präoperativ und bis zu 6 Monate nach der Operation betreut hatten.

Der Interviewleitfaden enthielt die Einstiegsfrage *„Bitte erzählen Sie mir, wie Sie die Begleitung der Patientinnen und Patienten, die von Ihnen im TPS betreut werden, erleben“* und setzte dann den Fokus auf das Erleben der

- Behandlung durch den TPS,
- Zusammenarbeit im TPS und
- Zusammenarbeit mit anderen Behandelnden außerhalb des TPS.

Die Fokusgruppeninterviews wurden nach tontechnischer Aufnahme entlang von vorab definierten Transkriptionsregeln [20] verschriftlicht und einer strukturierenden qualitativen Inhaltsanalyse nach Mayring [23] zugeführt. Die Analyseprozesse Codierung und Kategorisierung erfolgten mit der Software MAXQDA 6 (MAXQDA – VERBI, Berlin; [22]).

##### Perspektive der Patientinnen und Patienten.

Zur Erfassung der Perspektive der Patientinnen und Patienten standen deren Erleben bzw. Wahrnehmungen in Bezug auf die Umsetzung der nVF im Vordergrund. Diese Erhebung bzw. Auswertung sollte ermöglichen, die angestrebte Versorgung(squalität) rund um eine Operation aus Sicht der Nutzerinnen und Nutzer – neben primären und sekundären Endpunkten im Projekt POET-Pain – im Zusammenspiel mit den TPS-Teams qualitativ zu beschreiben.

Entlang der Forschungsfrage *„Wie haben Patientinnen und Patienten die Versorgung durch den TPS erlebt?*“ wurde ein halbstandardisierter Interviewleitfaden entwickelt, der für die Befragung in einem episodischen Interview eingesetzt wurde. Das episodische Interview wurde gewählt, da es den Interviewführenden ermöglicht, sehr nahe an einer Alltagskommunikation zu bleiben, indem eine Kombination aus Narration und Befragung im Interview erfolgen konnte [21]. Die Interviewführenden setzten nach Begriffsklärung zum TPS und der Einstiegsfrage *„Bitte erzählen Sie mir, was Sie im Kontakt mit dem ‚Transitional Pain Service‘ im Krankenhaus und nach Entlassung erlebt haben*“ gezielt weitere Erzählanreize, wie etwa:

- Spezifische Behandlung durch z. B. Ärztin/Arzt, Physiotherapeutin/Physiotherapeut, Pflegefachperson, Psychologin/Psychologe
- Behandlungsanteile – besonders hilfreich, weniger hilfreich
- Behandlungsanteile – Integration in den Alltag
- Bedingungen – förderlich, hinderlich
- Zusammenarbeit zwischen TPS und Regelversorgung

Für diese Datenerhebung wurden ausschließlich Patientinnen und Patienten der Studiengruppe „Risikotherapiegruppe“ eingeschlossen. Hierbei handelte es sich um Patientinnen und Patienten, bei denen das präoperative Screening im Projekt POET-Pain ein potenziell erhöhtes Risiko für CPSP ergeben hatte und die aufgrund dessen in die Gruppe „Behandlung durch den TPS“ randomisiert worden waren.

Aus jeder Klinik sollten zu 2 Erhebungszeitpunkten innerhalb der Interventionsphase (6. Monat und 9. Monat nach Interventionsstart) jeweils Interviewdaten von 2 Patientinnen und Patienten erhoben werden (Abb. 2). Zu den jeweiligen Zeitpunkten wurde das Studienpersonal in den Kliniken gebeten, Patientinnen und Patienten für die geplanten Telefoninterviews zu identifizieren, die der Teilnahme an einem zusätzlichen Telefoninterview im Rahmen der Studienaufklärung zugestimmt hatten. Die Ziehung der Stichprobe erfolgte durch die medizinischen Dokumentare in den Kliniken, die selbst keine Selektion getroffen hatten. Folgende Merkmale wurden entlang der Identifikationsnummern von Patientinnen und Patienten herangezogen:

- Geschlecht (männlich/weiblich)
- Alter (unter 51 Jahre, 51 Jahre und älter)
- Operationsdatum

Aus dieser pseudonymisierten Stichprobe wurden von der medizinischen Dokumentarin des wissenschaftlichen Teams jeweils 10 Patientinnen bzw. Patienten unter Berücksichtigung einer möglichst ausgeglichenen Verteilung der oben beschriebenen Merkmale ausgewählt und an die Kliniken zurückgemeldet, um für diese die Pseudonymisierung aufzuheben. Über eine datenschutzkonforme Plattform mit separater Passwortvergabe wurde die Übermittlung von personalisierten Kontaktdaten (Name und Telefonnummer) an das wissenschaftliche Team durchgeführt. Bei der Kontaktaufnahme mit den Patientinnen und Patienten wurde angestrebt, für die Interviews diese Merkmale über beide Erhebungszeitpunkt bzw. alle Kliniken homogen verteilt zu berücksichtigen (siehe Online-Zusatzmaterial 2: Merkmalsverteilung).

Die Interviews wurden tontechnisch aufgezeichnet. Die Auswertung der Einzelinterviews erfolgte – nach der Verschriftlichung entlang vorab definierter Transkriptionsregeln [20] – mittels inhaltlich strukturierender qualitativer Inhaltsanalyse [23]. Die Analyseprozesse Codierung und Kategorisierung erfolgten mit Computerunterstützung unter Anwendung der Software MAXQDA 6 (MAXQDA – VERBI, Berlin; [22]).

### Interpretation der Ergebnisse – Mixed-methods-Analyse

Wie bereits oben beschrieben (Abb. 1), erfolgte die Mixed-methods-Analyse zur Interpretation der Ergebnisse aufeinanderfolgend, da davon ausgegangen wurde, dass die qualitativen Ergebnisse (Phase II) ein vertiefendes Verständnis der quantitativen Ergebnisse (Phase I) liefern würden [19].

Die Erkenntnisse aus den beiden Phasen sollen in Joint-Displays abgebildet werden

In Abb. 3 ist das Prozessdiagramm des hier vorgestellten Explanatory-Sequential-Mixed-Methods-Designs (Zwei-Phasen-Design) gezeigt, das in Anlehnung an die Empfehlungen von Haynes-Brown u. Fetters [15] entwickelt wurde. Darin werden neben der Erhebungs- und Auswertungsphase der Datenerhebungsprozess inklusive der angestrebten Stichproben sowie auch die einzelnen Analyseprozesse dargestellt.

**Abb. 4:**
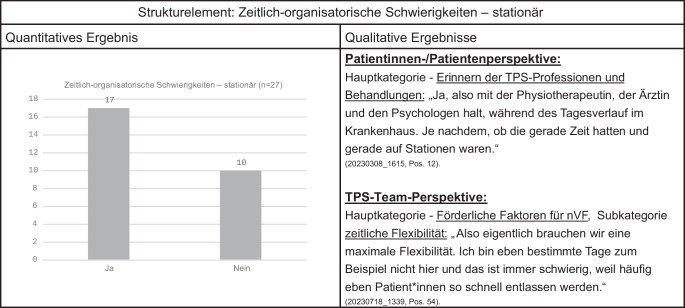
Joint-Display – Vertiefungsdisplay. *nVF* neue Versorgungsformen, *TPS* Transitional Pain Service

Für die detaillierte Darstellung und Nachvollziehbarkeit bei der Interpretation der Ergebnisse ist geplant, die Erkenntnisse aus den beiden Phasen in Joint-Displays, sogenannten Vertiefungsdisplays ([19]; Abb. 4), abzubilden [14, 15]. Diese Darstellungen ermöglichen die erweiterte Diskussion für ein vertiefendes Verständnis der quantitativen Daten zu den Strukturen und Prozessen der nVF, hier des TPS im Kontext der Begleiterhebung am Beispiel POET-Pain. Wie aus Abb. 4 beispielhaft ersichtlich, zeigt sich die Thematik der zeitlich-organisatorischen Schwierigkeiten in der stationären Phase sowohl in den quantitativen als auch in den qualitativen Daten. Diese Ergebnisse, hier exemplarisch im Joint-Display dargestellt (Abb. 4), bilden die Grundlage für die letzte Phase – die vertiefende Mixed-methods-Analyse.

## Resümee und Ausblick

Hinsichtlich einer Verbesserung der Gesundheitsversorgung bietet die Versorgungsforschung, in der gezielt wissenschaftliche Perspektiven untersucht werden, einen unverzichtbaren Nutzen. Dieser Nutzen liegt in der Lieferung notwendiger Daten, die eine evidenzbasierte Entscheidung und Analyse zur Verbesserung der Versorgung(squalität) ermöglichen [29]. Somit stehen ineffiziente Praktiken in der Erbringung von Gesundheitsleistungen im Kontrast zum effizienten Einsatz von Ressourcen, der es ermöglicht, die Gesundheitsleistung spezifisch an Bedarfe und Präferenzen von Patientinnen und Patienten anzupassen. Der großen Herausforderung der Versorgungsforschung, hier der Erfassung, Analyse und Interpretation der Vielschichtigkeit von Versorgungssystemen, kann mit den entsprechenden methodischen Herangehensweisen zur Erfassung komplexer Sachverhalte begegnet werden.

Mit der Struktur- und Prozessevaluation unter Anwendung des Explanatory-Sequential-Mixed-Methods-Designs und entlang einer RCT – wie hier am Beispiel des Projekts POET-Pain (Begleiterhebung) dargestellt – kann für die festgelegte komplexe Intervention (hier TPS) ein vertieftes Verständnis erreicht werden. Die Erkenntnisse beziehen sich sowohl auf das Verstehen der Alltagsabläufe in der Versorgung, unter anderem die interprofessionelle bzw. interdisziplinäre Zusammenarbeit, als auch auf das Erleben des Nutzens einer nVF für Patientinnen und Patienten. Für die Umsetzung solch komplexer nVF kann diese Methode dienlich sein, um förderliche wie hinderliche Faktoren zu identifizieren [5, 24].

Relevant für die Gewinnung derartiger Erkenntnisse ist die gezielte Konzeption einer Mixed-methods-Untersuchung. Das Vorgehen bedarf der Bereitstellung von Ressourcen für die Umsetzung des Explanatory-Sequential-Mixed-Methods-Designs, da die Durchführung sowohl quantitativer als auch qualitativer Analysen zusätzliche Zeit und Ressourcen benötigt. Zudem sind in der Planung spezifische Kompetenzanforderungen an die Forschenden zu stellen, etwa hinsichtlich der Methodenkompetenz und Erfahrung in der quantitativen und qualitativen bzw. in der Mixed-methods-Forschung, da neben der umfänglichen Datenerhebung die Komplexität der Datenanalyse und -integration methodisch anspruchsvoll ist [6].

Mit Blick auf eine mögliche Implementierung komplexer Interventionen in die Regelversorgung kann mit der gezielten Erfassung der zweiten Säule (Gegenstand) der Versorgungsforschung ein umfassenderes und tieferes Verständnis der Versorgungsprozesse und gegebenenfalls der Versorgungsergebnisse erlangt werden. Denn die ergänzende Beschreibung lässt eine optimierende Anpassung der nVF zu, die dann in weiterer Folge eine erfolgreiche evidenzbasierte und patientinnen- bzw. patientenzentrierte Gesundheitsversorgung unterstützt.

## Fazit für die Praxis

- Die detaillierte Erforschung des Gegenstands als eine Säule in der Versorgungsforschung liefert einen detaillierten Einblick in die doppelte Komplexität: die neue Gesundheitsleistung und deren Kontext.
- Das Explanatory-Sequential-Mixed-Methods-Design liefert mit aufeinander folgenden Datenerhebungen und -analysen vertiefende Erkenntnisse zu komplexen Interventionen.
- Die Darstellung von Ergebnissen in Joint-Displays ermöglicht eine nachvollziehbare Interpretation der Kombination aus quantitativen und qualitativen Datensätzen.
- Die gezielte Planung einer Struktur- und Prozessevaluation als Begleiterhebung in Studien zur Umsetzung neuer Versorgungsformen kann zur Verbesserung der Versorgungsqualität beitragen.

## Supplementary Information

Online-Zusatzmaterial 1: Erhebungsbogen

Online-Zusatzmaterial 2: Merkmalsverteilung
