# Supplementary material for: Structure and process evaluation of complex interventions in pain therapy: Description of a methodological approach using the example of POET-Pain
Source: Schmerz. 2024 Dec 10;39(1):35–42. [Article in German] doi: 10.1007/s00482-024-00850-w (PMC11785641; doi:10.1007/s00482-024-00850-w)
Supplement: Supplementary file 1 — Online-Zusatzmaterial 1: Erhebungsbogen [file 482_2024_850_MOESM1_ESM.pdf]

Struktur- und Prozessevaluation

Die folgenden Fragen beziehen sich auf Angaben zu Ihrer Profession und Ihrem Anstellungsverhältnis im TPS-Team.

1. Bitte kreuzen Sie Ihre **Berufsgruppe** innerhalb des TPS-Teams an:
   - ☐ ärztliche Profession
   - ☐ physiotherapeutische Profession
   - ☐ psychologische Profession
   - ☐ pflegerische Profession
   - ☐ Sonstige (bitte benennen):

2. Bitte nennen Sie Ihre **tarifliche Einordnung** (z.B. E9):

3. Bitte geben Sie **Ihren Arbeitszeitumfang / Ihr Vollzeitäquivalent** (VZÄ) im TPS-Team an:
   - ☐ 100% VZÄ
   - ☐ 75% VZÄ
   - ☐ 50% VZÄ
   - ☐ 25% VZÄ
   - ☐ Sonstiges (bitte benennen):

4. Ist diese Stelle im TPS-Team auf mehrere Personen **aufgeteilt?**
   - ☐ Ja
   - ☐ Nein

   Wenn ja: Auf wie viele Personen? (bitte benennen):

5. Ist ein **gleichzeitiger Einsatz** in einer anderen Funktion (z.B. auch im Akutschmerzdienst) vorhanden?
   - ☐ Ja – bitte führen Sie Funktion und VZÄ aus:
   - ☐ Nein
   - ☐ Kann keine Aussage treffen

6. Gibt es für Ihre Stelle eine **Stellvertretung** für Krankheits- und Urlaubsphasen im TPS-Team?
   ☐ Ja – bitte führen Sie Funktion und VZÄ aus:
   ☐ Nein
   ☐ Kann keine Aussage treffen

   Wenn ja: Gibt es mehr als eine Vertretung?
   ☐ Ja
   ☐ Nein
   Wenn ja, wie viele Vertretungen gibt es? (bitte benennen):

Die folgenden Fragen beziehen sich auf räumliche und technische Voraussetzungen, die Sie als Mitglied des TPS-Teams haben.

7. Sind geeignete **Räumlichkeiten** zur störungsfreien Durchführung des **Patient/innenkontakts** vorhanden?
   ☐ Ja
   ☐ Nein, vorwiegend im Patient/innen-Zimmer
   ☐ Nein, vorwiegend auf dem Stationsflur
   ☐ Sonstiges (bitte benennen):

8. Ist ein separater **Raum** für die Durchführung der **TPS-Teambesprechungen** vorhanden?
   ☐ Ja
   ☐ Nein
   ☐ Sonstiges (bitte benennen):

9. Ist die technische Ausstattung in Form eines **mobilen Telefons** vorhanden?
   ☐ Ja, ein Gerät für jedes TPS-Mitglied (inkl. Vertretungen)
   ☐ Ja, ein Gerät für jede TPS-Profession (max. 4 Geräte)
   ☐ Nein

☐ Sonstiges (bitte benennen):

10. Ist die technische Ausstattung in Form eines **PCs** vorhanden?
   - ☐ Ja, ein Gerät für jedes TPS-Mitglied (inkl. Vertretungen)
   - ☐ Ja, ein Gerät für jede TPS-Profession (max. 4 Geräte)
   - ☐ Nein
   - ☐ Sonstiges (bitte benennen):

11. Ist die technische Ausstattung in Form eines **Laptops** vorhanden?
   - ☐ Ja, ein Gerät für jedes TPS-Mitglied (inkl. Vertretungen)
   - ☐ Ja, ein Gerät für jede TPS-Profession (max. 4 Geräte)
   - ☐ Nein
   - ☐ Sonstiges (bitte benennen):

12. Ist die technische Ausstattung in Form eines **Tablets** vorhanden?
   - ☐ Ja, ein Gerät für jedes TPS-Mitglied (inkl. Vertretungen)
   - ☐ Ja, ein Gerät für jede TPS-Profession (max. 4 Geräte)
   - ☐ Nein
   - ☐ Sonstiges (bitte benennen):

13. Ist die technische Ausstattung in Form von **WLAN** (für App-Nutzung) vorhanden?
   - ☐ Ja
   - ☐ Nein
   - ☐ teilweise (bitte benennen):
   - ☐ Sonstiges (bitte benennen):

14. Welches Vorgehen wird zur **Erhebung und Dokumentation der Patient/innen Kontakte** gegenwärtig vorwiegend genutzt?
   - ☐ TPS-App
   - ☐ beschreibbare PDF-Dokumente
   - ☐ sonstiges Vorgehen (bitte benennen):

Die folgenden Fragen beziehen sich auf die Organisation von Aufgaben des TPS-Teams.

15. Ist neben der Aufgabendefinition im übergeordneten Handbuch eine **schriftliche Stellenbeschreibung für Ihre Stelle im TPS** vorhanden?
    - ☐ Ja
    - ☐ Nein
    - ☐ Sonstiges (bitte benennen):

16. Gibt es **zeitlich-organisatorische Schwierigkeiten** einen Termin für die Patient/innenkontakte zu vereinbaren?

    a. Prästationär
    - ☐ Ja
    - ☐ Nein

    Wenn ja, welche Schwierigkeiten (bitte benennen):

    b. Stationär
    - ☐ Ja
    - ☐ Nein

    Wenn ja, welche Schwierigkeiten (bitte benennen):

    c. Ambulant (aktuell bis 1. postoperativer Monat)
    - ☐ Ja
    - ☐ Nein

    Wenn ja, welche Schwierigkeiten (bitte benennen):

17. Wie erfolgt die **Abstimmung** der individuellen, professionsspezifischen **Therapieempfehlungen im TPS-Team**?
    - ☐ durch Eingabe der individuellen professionsspezifischen Risikoprofile

- ☐ durch individuelle Fallbesprechung
- ☐ durch Konsens
- ☐ durch Mehrheitsbeschluss
- ☐ durch Festlegung einer Profession im TPS-Team
- ☐ als gemeinsame Erarbeitung im Rahmen der TPS-Teamsitzung
- ☐ in Verantwortung der ärztlichen Profession
- ☐ Sonstiges (bitte benennen):

18. Welche Regelungen zu **Schmerztherapiekonzepten** sind in der Klinik vorhanden und werden in der Versorgung durch das TPS-Team berücksichtigt?
    - ☐ Regelungen entlang aktueller Leitlinien
    - ☐ Klinikinterne SOP
    - ☐ Regelungen des Akutschmerzdienstes
    - ☐ Regelungen des chronischen Schmerzdienstes
    - ☐ Regelungen der jeweiligen Fachabteilung
    - ☐ sonstige Regelungen (bitte benennen):
    - ☐ keine Regelungen vorhanden

Die folgenden Fragen beziehen sich auf die Abstimmung und Kommunikation aller Professionen im TPS-Team.

19. Welche Profession übernimmt **die Koordination** im TPS-Team?
    - ☐ ärztliche Profession
    - ☐ physiotherapeutische Profession
    - ☐ pflegerische Profession
    - ☐ psychologische Profession
    - ☐ Sonstige (bitte benennen):

20. Welche **Kommunikationswege und -medien** werden im TPS-Team genutzt?
    (Mehrfachantworten möglich)
    - ☐ Arbeitslisten
    - ☐ Gesprächsnotizen
    - ☐ persönliche E-Mails

- ☐ Gruppen-E-Mails
- ☐ Telefon
- ☐ Outlook-Kalender (gemeinsame Nutzung)
- ☐ TPS-App
- ☐ persönliche Absprachen
- ☐ Sonstiges (bitte benennen):

Die folgenden Fragen beziehen sich auf die regelmäßigen TPS-Teamsitzungen.

21a. **Wann** finden vorwiegend die regelmäßigen TPS-Teamsitzungen statt?

(Mehrfachantwort möglich)

- ☐ Montagvormittags
- ☐ Montagnachmittags
- ☐ Dienstagvormittags
- ☐ Dienstagnachmittags
- ☐ Mittwochvormittags
- ☐ Mittwochnachmittags
- ☐ Donnerstagvormittags
- ☐ Donnerstagnachmittags
- ☐ Freitagvormittags
- ☐ Freitagnachmittags
- ☐ Keine festen Tage festgelegt
- ☐ Sonstiges (bitte benennen):

21 b. **Wie häufig** finden regelmäßige TPS-Teamsitzungen statt?

- ☐ 1x wöchentlich
- ☐ 2x wöchentlich
- ☐ 3x wöchentlich
- ☐ Sonstiges (bitte benennen):

21c. **Wie lange** dauern durchschnittlich die regelmäßigen TPS-Teamsitzungen?

- ☐ weniger als 30 Minuten
- ☐ 1 Stunde

☐ 1,5 Stunden
☐ 2 Stunden
☐ 2,5 Stunden
☐ Sonstiges (bitte benennen):

21d. Ist die **Dauer** der regelmäßigen TPS-Teamsitzungen **ausreichend** zur Besprechung und Dokumentation der Patient/innen?

☐ Ja
☐ Nein (bitte begründen):

21e. Wie wird mit der **Abwesenheit Ihrer Profession** in der TPS-Teamsitzung verfahren? Bitte beschreiben Sie dies kurz:

21f. Wie gestaltet sich die **TPS-Teamsitzung** für die Betreuung der Patient/innen in der **ambulanten (poststationären) Phase**? Bitte beschreiben Sie dies kurz:

22. Stehen Sie als TPS-Mitglied regelmäßig in **Kontakt mit Kolleg/innen auf den Stationen** rund um die Vermittlung der Therapieempfehlung?

☐ Nein
☐ Ja, täglich
☐ Ja, wöchentlich
☐ Ja, nach jeder TPS-Teamsitzung
☐ Ja, zu besonderen Aspekten (bitte benennen):
☐ Sonstige Kontakte (bitte benennen):

Wenn ja: Mit wem stehen Sie regelmäßig in Kontakt?
(Mehrfachantwort möglich)

☐ Stationsärztinnen/-ärzten
☐ Pflegenden
☐ Physiotherapeut/innen
☐ Psycholog/innen

☐ Sonstige (bitte benennen):

Die folgenden Fragen beziehen sich auf die Regelungen zur Vermittlung der abgestimmten Therapieempfehlungen an die ambulant versorgten Patient/innen.

23a. **Welche Profession** übernimmt die Vermittlung der professionsspezifischen Therapieempfehlungen an die Patient/innen?

(Mehrfachantworten möglich)

☐ ärztliche Profession
☐ physiotherapeutische Profession
☐ pflegerische Profession
☐ psychologische Profession
☐ Sonstiges (bitte benennen):

23b. In **welcher Form** erfolgt die Vermittlung der professionsspezifischen Therapieempfehlungen an die Patient/innen?

(Mehrfachantworten möglich)

☐ persönlich
☐ telefonisch
☐ schriftlich
☐ unklar
☐ Sonstiges (bitte benennen):

24. Welche Regelungen **zur poststationären Kontaktaufnahme** zwischen TPS-Team und Patient/innen gibt es?

(Mehrfachantworten möglich)

☐ 1. poststationärer Termin wird fest vereinbart
☐ telefonische Kontaktaufnahme für Nachbesprechung und Terminbestätigung
☐ Patient/innen bekommen Kontaktdaten für Bedarfsfall ausgehändigt
☐ es werden 1-3 Termine geplant (mind. 1 Termin davon in Präsenz)
☐ Sonstiges (bitte benennen):

25. **Wie** können Patient/innen das TPS-Team in der ambulanten Phase **erreichen**?
(Mehrfachantworten möglich)
- ☐ persönlich vor Ort (bitte Zeiten benennen):
- ☐ per Telefon (bitte Zeiten benennen):
- ☐ per E-Mail
- ☐ per Videokonferenz
- ☐ Sonstiges (bitte benennen):

Die folgenden Fragen beziehen sich auf die Vermittlung der abgestimmten professionsspezifischen Therapieempfehlungen an die ambulanten Weiterbehandler/innen, also nach Entlassung der Patient/innen aus der TPS-Behandlung.

26a. **Welche Profession** übernimmt die Vermittlung der Therapieempfehlungen?
(Mehrfachantworten möglich)
- ☐ ärztliche Profession
- ☐ physiotherapeutische Profession
- ☐ pflegerische Profession
- ☐ psychologische Profession
- ☐ Sonstiges (bitte benennen):

26b. **An welche Institutionen /Fachpersonen** werden die Therapieempfehlungen vermittelt?
(Mehrfachantworten möglich)
- ☐ Hausarzt/-ärztin
- ☐ Facharzt/-ärztin
- ☐ Rehabilitationseinrichtung
- ☐ Pflegedienst
- ☐ Physiotherapeut/-in
- ☐ Psychologe/-in
- ☐ Sonstige (bitte benennen):

26c. In **welcher Form** erfolgt die Vermittlung der Therapieempfehlungen?

(Mehrfachantworten möglich)

☐ separater Entlassbrief

☐ Therapieempfehlungen sind Teil des Entlassbriefes

☐ Zwischenbericht zur TPS Behandlung

☐ persönliche Mitteilung an die ambulanten Mitbehandler/innen

☐ persönliche Mitteilung an den/die Patienten/in

☐ Sonstiges (bitte benennen):

26d. **Wie** erfolgt die Vermittlung der Therapieempfehlung?

(Mehrfachantworten möglich)

☐ persönlich

☐ telefonisch

☐ schriftlich

☐ unklar

☐ Sonstiges (bitte benennen):
