# Supplementary material for: Structure and process evaluation of complex interventions in pain therapy: Description of a methodological approach using the example of POET-Pain
Source: Schmerz. 2024 Dec 10;39(1):35–42. [Article in German] doi: 10.1007/s00482-024-00850-w (PMC11785641; doi:10.1007/s00482-024-00850-w)
Supplement: Supplementary file 2 — Online-Zusatzmaterial 2: Merkmalsverteilung [file 482_2024_850_MOESM2_ESM.pdf]

Kliniken / **Merkmalsverteilung (1. Erhebung)**	**K 1**	**K 2**	**K 3**	**K 4**	**K 5**	**K 6**
Frau < 51 Jahre	X					
Frau ≥ 51 Jahre und älter						X
Mann < 51 Jahre		X				
Mann ≥ 51 Jahre und älter					X	
Patient/innen mit vorbestehenden Schmerz (> 3 Monate)			X			
				X		
Eine Woche nach Entlassung				X		
			X			
3 Monate nach erster Leistung TPS					X	
		X				
6 Monate nach erster Leistung TPS						X
	X					
Kliniken / **Merkmalsverteilung (2. Erhebung)**	**K 1**	**K 2**	**K 3**	**K 4**	**K 5**	**K 6**
Frau < 51 Jahre						X
Frau ≥ 51 Jahre und älter	X					
Mann < 51 Jahre					X	
Mann ≥ 51 Jahre und älter		X				
Patient/innen mit vorbestehenden Schmerz (> 3 Monate)				X		
			X			
Eine Woche nach Entlassung			X			
				X		
3 Monate nach erster Leistung TPS		X				
					X	
6 Monate nach erster Leistung TPS	X					
						X

Supplement 2 Merkmalsverteilung
